# A redetermination of the structure of poly[[μ_4_-(*R*)-2-ammonio-3-sulfonato­propano­ato]aqua­sodium], originally reported as poly[[μ_7_-l-cysteato(2−)]disodium]

**DOI:** 10.1107/S1600536812009683

**Published:** 2012-03-10

**Authors:** I. David Brown

**Affiliations:** a Brockhouse Institute for Materials Research, McMaster University Hamilton, Ontario, Canada L8S 4M1

## Abstract

The structure originally reported as poly[[μ_7_-l-cysteato(2−)]disodium], [Na_2_(C_3_H_5_NO_5_S)]_*n*_ [Liu (2002). *Acta Cryst*. E**67**, m1346–m1347], has been redetermined with one of the sodium atoms replaced with a water mol­ecule and an additional proton attached to the amine group, resulting in the revised formula [Na{CO_2_CH(CH_2_SO_3_)NH_3_}(H_2_O)]_*n*_. The agreement index, *wR*, has been reduced from 0.159 to 0.087 and the global instability index from 0.56 vu (valence units) to the acceptable value of 0.11 vu.

## Related literature

The original structure determination of this compound was reported by Liu (2011 ▶). The bond-valence methods are described in Brown (2002 ▶).
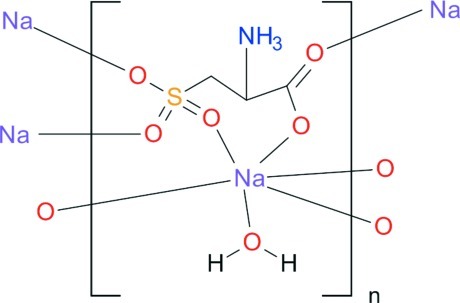


## Experimental

### 

#### Crystal data

[Na(C_3_H_6_NO_5_S)(H_2_O)]
*M* *_r_* = 209.15
Monoclinic, 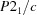

*a* = 5.7574 (12) Å
*b* = 11.875 (2) Å
*c* = 11.691 (3) Å
β = 109.15 (3)°
*V* = 755.1 (3) Å^3^
*Z* = 4
Mo *K*α radiation
μ = 0.48 mm^−1^
*T* = 298 K
0.24 × 0.22 × 0.20 mm

#### Data collection

Rigaku SCX-Mini CCD diffractometer
Absorption correction: multi-scan (*ABSCOR*; Higashi, 1995 ▶) *T*_min_ = 0.894, *T*_max_ = 0.911
7845 measured reflections
1740 independent reflections
1463 reflections with *I* > 2Σ(*I*)
*R*_int_ = 0.044

#### Refinement

*R*[*F* ^2^ > 2σ(*F* ^2^)] = 0.038
*wR*(*F* ^2^) = 0.087
*S* = 1.10
1740 reflections
118 parameters
2 restraints
H atoms treated by a mixture of independent and constrained refinement
Δρ_max_ = 0.39 e Å^−3^
Δρ_min_ = −0.35 e Å^−3^

### 

Data collection: *PROCESS-AUTO* (Rigaku, 1998 ▶); cell refinement: *PROCESS-AUTO*; data reduction: *CrystalStructure* (Rigaku/MSC, 2002 ▶); method used to solve structure: coordinates taken from the previous refinement (Liu, 2011 ▶); program(s) used to refine structure: *SHELXL97* (Sheldrick, 2008 ▶); molecular graphics: *Mercury* (Macrae *et al.*, 2006 ▶); software used to prepare material for publication: *publCIF* (Westrip, 2010 ▶).

## Supplementary Material

Crystal structure: contains datablock(s) global, I. DOI: 10.1107/S1600536812009683/sj5205sup1.cif

Structure factors: contains datablock(s) I. DOI: 10.1107/S1600536812009683/sj5205Isup2.hkl

Additional supplementary materials:  crystallographic information; 3D view; checkCIF report

## Figures and Tables

**Table 1:** Selected bond lengths (Å)

Na1—O4^i^	2.3512 (19)
Na1—O5^ii^	2.3619 (19)
Na1—O3	2.4183 (18)
Na1—O2^iii^	2.4272 (19)
Na1—O1*W*	2.450 (2)
Na1—O1	2.4778 (18)

Symmetry codes: (i) 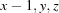; (ii) 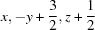; (iii) 
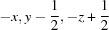
.

**Table 2:** Hydrogen-bond geometry (Å, °)

*D*—H⋯*A*	*D*—H	H⋯*A*	*D*⋯*A*	*D*—H⋯*A*
N1—H1*A*⋯O1*W*^iv^	0.89	2.16	2.981 (3)	153
N1—H1*B*⋯O1^v^	0.89	1.97	2.842 (2)	166
N1—H1*C*⋯O2^iv^	0.89	1.88	2.766 (2)	173
O1*W*—H1*WA*⋯O3^ii^	0.85 (1)	2.10 (1)	2.912 (3)	160 (4)
O1*W*—H1*WB*⋯O5^vi^	0.85 (1)	2.08 (1)	2.930 (2)	178 (3)

Symmetry codes: (ii) 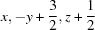; (iv) 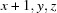; (v) 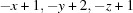; (vi) 
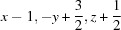
.

**Fig. 1.**
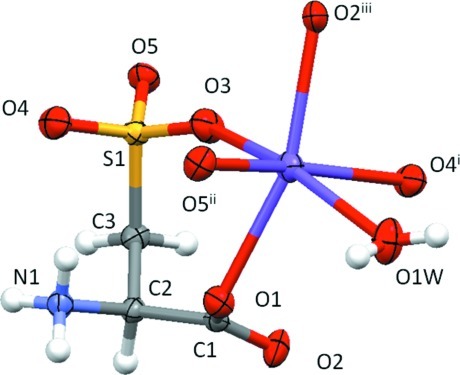
The environment of the Na+ cation with atomic displacement parameters drawn at the 50% probability level. [Symmetry codes: (i) x - 1, y, z; (ii) x, -y + 3/2, z + 1/2; (iii) -x, y - 1/2, -z + 1/2.]

## supplementary crystallographic information

### Comment

In a recent issue of this journal, Liu (2011) reported the structure of the title compound, but a close examination of this structure shows a serious problem with the environment of one of the sodium atoms, Na2. In the Comment the author describes this atom as 'tetracoordinated within an NO_3_ coordination sphere. The Na^+^ ion binds to the amino N atom, to one of the O atom of the carboxylic residue and to two O atoms of the sulfonate group in a distorted tetrahedral arrangement'. The Comment does not point out that the atomic displacement parameter of Na2 is almost three times larger than the next largest atomic displacement parameter, nor does it point out that the lengths of the four bonds around Na2 all lie in the range 2.90 to 3.03 Å, distances whose bond valence sum of 0.21 vu (valence unit) indicates that they are much longer than would be expected for a four-coordinate sodium cation. The global instability index [root mean square deviation of the bond valence sums of all atoms from their atomic valence, (Brown, 2002)] is 0.56 vu, much higher than the generally accepted limit of 0.20 vu for a stable structure. The environment of Na2 is, however, one that would be expected for a water molecule that forms moderate to weak hydrogen bonds. An additional hydrogen ion is required for charge neutrality, but protonating the water molecule is unlikely as this would require much shorter hydrogen bonds than are observed for this site, but protonating the amine group would not only increase the bond valence sum around the nitrogen from 2.51 vu to a value closer to the expected 3.00 vu, it would also result in an N—H bond positioned to form a hydrogen bond with the water molecule.

This proposed model has been refined and is reported in this paper. The bond valence sum of 0.21 vu around the original Na2 has been replaced by a sum of 1.94 vu around the oxygen of water, and the sum around the N1 atom has been increased to 3.17 vu. Moreover, Na1 now has a meaningful octahedral environment (Fig. 1) with a Na1—O1W contact of 2.450 (2) Å instead of a Na1—Na2 contact as in the original model.

Based on the rerefinement of the structure, this crystal must be reformulated as sodium (*R*)-2-ammonium-3-sulfopropanoate monohydrate, Na(CO_2_CH(CH_2_SO_3_)NH_3_)(H_2_O).

### Experimental

The preparation of the compound is detailed in the original report (Liu, 2011).

### Refinement

The structure was refined using the original diffraction measurements of Liu (2011). For re-refinement of the original model, all H atoms were removed in the first refinement cycle and the doubtful atom Na2 replaced by an O atom (O1W). All H atoms were discernible from difference maps. H atoms attached to C atoms were finally included in calculated positions using a riding model with bond lengths C—H = 0.97 or 0.98 Å and *U*_iso_(H) = 1.2 times *U*_eq_(C); H atoms attached to the ammonium group were constrained to bond lengths N—H = 0.89 Å with *U*_iso_(H) = 1.5 times *U*_eq_(N). The water H atoms were restrained to bond lengths of 0.85 (1) Å.

### Crystal data

**Table:**

[Na(C_3_H_6_NO_5_S)(H_2_O)]	*F*(000) = 432
*M**_r_* = 209.15	*D*_x_ = 1.840 Mg m^−^^3^
Monoclinic, *P*2_1_/*c*	Mo *K*α radiation, λ = 0.71073 Å
Hall symbol: -P 2ybc	Cell parameters from 7309 reflections
*a* = 5.7574 (12) Å	θ = 3.4–27.5°
*b* = 11.875 (2) Å	µ = 0.48 mm^−^^1^
*c* = 11.691 (3) Å	*T* = 298 K
β = 109.15 (3)°	Prism, colourless
*V* = 755.1 (3) Å^3^	0.24 × 0.22 × 0.20 mm
*Z* = 4	

### Data collection

**Table:**

Rigaku SCX-Mini CCD diffractometer	1740 independent reflections
Radiation source: fine-focus sealed tube	1463 reflections with *I* > 2Σ(*I*)
Graphite monochromator	*R*_int_ = 0.044
ω scans	θ_max_ = 27.5°, θ_min_ = 3.4°
Absorption correction: multi-scan (*ABSCOR*; Higashi, 1995)	*h* = −7→7
*T*_min_ = 0.894, *T*_max_ = 0.911	*k* = −15→15
7845 measured reflections	*l* = −15→15

### Refinement

**Table:**

Refinement on *F*^2^	Primary atom site location: structure-invariant direct methods
Least-squares matrix: full	Secondary atom site location: difference Fourier map
*R*[*F*^2^ > 2σ(*F*^2^)] = 0.038	Hydrogen site location: inferred from neighbouring sites
*wR*(*F*^2^) = 0.087	H atoms treated by a mixture of independent and constrained refinement
*S* = 1.10	*w* = 1/[σ^2^(*F*_o_^2^) + (0.0337*P*)^2^ + 0.5281*P*] where *P* = (*F*_o_^2^ + 2*F*_c_^2^)/3
1740 reflections	(Δ/σ)_max_ = 0.001
118 parameters	Δρ_max_ = 0.39 e Å^−^^3^
2 restraints	Δρ_min_ = −0.35 e Å^−^^3^

### Special details

**Table:**

Geometry. All e.s.d.'s (except the e.s.d. in the dihedral angle between two l.s. planes) are estimated using the full covariance matrix. The cell e.s.d.'s are taken into account individually in the estimation of e.s.d.'s in distances, angles and torsion angles; correlations between e.s.d.'s in cell parameters are only used when they are defined by crystal symmetry. An approximate (isotropic) treatment of cell e.s.d.'s is used for estimating e.s.d.'s involving l.s. planes.
Refinement. Refinement of *F*^2^ against ALL reflections. The weighted *R*-factor *wR* and goodness of fit *S* are based on *F*^2^, conventional *R*-factors *R* are based on *F*, with *F* set to zero for negative *F*^2^. The threshold expression of *F*^2^ > σ(*F*^2^) is used only for calculating *R*-factors(gt) *etc*. and is not relevant to the choice of reflections for refinement. *R*-factors based on *F*^2^ are statistically about twice as large as those based on *F*, and *R*- factors based on ALL data will be even larger.

### Fractional atomic coordinates and isotropic or equivalent isotropic displacement parameters (Å^2^)

**Table:**

	*x*	*y*	*z*	*U*_iso_*/*U*_eq_	
Na1	0.04743 (15)	0.74874 (7)	0.32550 (8)	0.0227 (2)	
C1	0.2113 (4)	1.00587 (18)	0.31408 (19)	0.0187 (4)	
C2	0.4306 (4)	1.03306 (18)	0.27159 (19)	0.0183 (4)	
H2	0.4392	1.1153	0.2677	0.022*	
C3	0.3962 (4)	0.98919 (18)	0.14342 (19)	0.0202 (5)	
H3A	0.5201	1.0241	0.1155	0.024*	
H3B	0.2371	1.0146	0.0905	0.024*	
N1	0.6664 (3)	0.99540 (16)	0.36077 (16)	0.0229 (4)	
H1A	0.6638	0.9210	0.3694	0.034*	
H1B	0.6869	1.0284	0.4318	0.034*	
H1C	0.7900	1.0143	0.3347	0.034*	
O1	0.2380 (3)	0.93424 (13)	0.39576 (13)	0.0244 (4)	
O2	0.0199 (3)	1.06104 (14)	0.26136 (16)	0.0294 (4)	
O3	0.2321 (3)	0.78831 (14)	0.17151 (15)	0.0310 (4)	
O4	0.6636 (3)	0.80928 (14)	0.19436 (15)	0.0300 (4)	
O5	0.3578 (3)	0.82358 (14)	−0.00367 (13)	0.0251 (4)	
S1	0.41302 (9)	0.84075 (4)	0.12587 (5)	0.01761 (15)	
O1W	−0.1946 (4)	0.76543 (17)	0.46236 (17)	0.0356 (4)	
H1WA	−0.090 (5)	0.759 (4)	0.5329 (15)	0.090 (15)*	
H1WB	−0.326 (3)	0.740 (3)	0.471 (3)	0.058 (10)*	

### Atomic displacement parameters (Å^2^)

**Table:**

	*U*^11^	*U*^22^	*U*^33^	*U*^12^	*U*^13^	*U*^23^
Na1	0.0214 (5)	0.0242 (5)	0.0216 (5)	0.0005 (4)	0.0058 (4)	0.0015 (4)
C1	0.0195 (11)	0.0171 (10)	0.0209 (11)	−0.0036 (9)	0.0087 (9)	−0.0060 (9)
C2	0.0178 (10)	0.0169 (10)	0.0221 (11)	0.0000 (8)	0.0091 (9)	−0.0003 (8)
C3	0.0228 (11)	0.0187 (11)	0.0209 (11)	0.0011 (9)	0.0094 (9)	0.0013 (9)
N1	0.0174 (9)	0.0297 (10)	0.0223 (10)	−0.0022 (8)	0.0073 (8)	−0.0045 (8)
O1	0.0275 (9)	0.0259 (8)	0.0216 (8)	−0.0031 (7)	0.0105 (7)	0.0012 (7)
O2	0.0195 (8)	0.0306 (9)	0.0411 (10)	0.0060 (7)	0.0139 (7)	0.0091 (8)
O3	0.0366 (10)	0.0311 (9)	0.0319 (9)	−0.0106 (8)	0.0203 (8)	−0.0026 (7)
O4	0.0256 (9)	0.0286 (9)	0.0291 (9)	0.0075 (7)	0.0000 (7)	−0.0007 (7)
O5	0.0264 (8)	0.0309 (9)	0.0184 (8)	−0.0045 (7)	0.0079 (7)	−0.0053 (6)
S1	0.0193 (3)	0.0179 (3)	0.0160 (3)	−0.0013 (2)	0.0063 (2)	−0.0006 (2)
O1W	0.0274 (10)	0.0517 (12)	0.0302 (10)	0.0023 (9)	0.0127 (9)	0.0119 (9)

### Geometric parameters (Å, º)

**Table:**

Na1—O4^i^	2.3512 (19)	C3—S1	1.781 (2)
Na1—O5^ii^	2.3619 (19)	C3—H3A	0.9700
Na1—O3	2.4183 (18)	C3—H3B	0.9700
Na1—O2^iii^	2.4272 (19)	N1—H1A	0.8900
Na1—O1W	2.450 (2)	N1—H1B	0.8900
Na1—O1	2.4778 (18)	N1—H1C	0.8900
C1—O1	1.250 (3)	O3—S1	1.4567 (16)
C1—O2	1.256 (3)	O4—S1	1.4505 (17)
C1—C2	1.535 (3)	O5—S1	1.4561 (16)
C2—N1	1.484 (3)	O1W—H1WA	0.8499 (11)
C2—C3	1.537 (3)	O1W—H1WB	0.8500 (11)
C2—H2	0.9800		
			
O4^i^—Na1—O5^ii^	162.02 (7)	C2—C3—H3A	108.1
O4^i^—Na1—O3	90.22 (7)	S1—C3—H3A	108.1
O5^ii^—Na1—O3	107.74 (7)	C2—C3—H3B	108.1
O4^i^—Na1—O2^iii^	91.21 (7)	S1—C3—H3B	108.1
O5^ii^—Na1—O2^iii^	89.57 (7)	H3A—C3—H3B	107.3
O3—Na1—O2^iii^	85.17 (6)	C2—N1—H1A	109.5
O4^i^—Na1—O1W	77.69 (7)	C2—N1—H1B	109.5
O5^ii^—Na1—O1W	84.94 (7)	H1A—N1—H1B	109.5
O3—Na1—O1W	162.46 (7)	C2—N1—H1C	109.5
O2^iii^—Na1—O1W	107.49 (7)	H1A—N1—H1C	109.5
O4^i^—Na1—O1	99.41 (7)	H1B—N1—H1C	109.5
O5^ii^—Na1—O1	85.01 (6)	C1—O1—Na1	114.94 (13)
O3—Na1—O1	79.58 (6)	C1—O2—Na1^iv^	132.70 (14)
O2^iii^—Na1—O1	161.39 (6)	S1—O3—Na1	153.84 (11)
O1W—Na1—O1	89.79 (7)	S1—O4—Na1^v^	172.12 (12)
O1—C1—O2	126.7 (2)	S1—O5—Na1^vi^	140.97 (10)
O1—C1—C2	118.85 (19)	O4—S1—O5	112.10 (10)
O2—C1—C2	114.45 (18)	O4—S1—O3	112.88 (11)
N1—C2—C1	111.67 (17)	O5—S1—O3	112.51 (10)
N1—C2—C3	112.25 (17)	O4—S1—C3	105.86 (10)
C1—C2—C3	112.80 (17)	O5—S1—C3	104.88 (10)
N1—C2—H2	106.5	O3—S1—C3	107.96 (10)
C1—C2—H2	106.5	Na1—O1W—H1WA	105 (3)
C3—C2—H2	106.5	Na1—O1W—H1WB	140 (2)
C2—C3—S1	116.96 (15)	H1WA—O1W—H1WB	103 (3)

Symmetry codes: (i) *x*−1, *y*, *z*; (ii) *x*, −*y*+3/2, *z*+1/2; (iii) −*x*, *y*−1/2, −*z*+1/2; (iv) −*x*, *y*+1/2, −*z*+1/2; (v) *x*+1, *y*, *z*; (vi) *x*, −*y*+3/2, *z*−1/2.

### Hydrogen-bond geometry (Å, º)

**Table:**

*D*—H···*A*	*D*—H	H···*A*	*D*···*A*	*D*—H···*A*
N1—H1*A*···O1*W*^v^	0.89	2.16	2.981 (3)	153
N1—H1*B*···O1^vii^	0.89	1.97	2.842 (2)	166
N1—H1*C*···O2^v^	0.89	1.88	2.766 (2)	173
O1*W*—H1*WA*···O3^ii^	0.85 (1)	2.10 (1)	2.912 (3)	160 (4)
O1*W*—H1*WB*···O5^viii^	0.85 (1)	2.08 (1)	2.930 (2)	178 (3)

Symmetry codes: (ii) *x*, −*y*+3/2, *z*+1/2; (v) *x*+1, *y*, *z*; (vii) −*x*+1, −*y*+2, −*z*+1; (viii) *x*−1, −*y*+3/2, *z*+1/2.
